# Frank Howard Davis, M. D.

**Published:** 1880-09

**Authors:** 


DIED, at 9:30 a.m. of August 17th, 1880, at his residence, 291 Huron Street, Chicago, FRANK HOWARD DAVIS, M.D., aged 32 years, two months and twelve days.

The deceased was born in the city of New York, June 5, 1848, was taken by his parents, Nathan S. and Anna Maria Davis, to Chicago in September, 1849, where he lived until his death. He received the earlier part of his education in the select schools of this city, and entered the University of Michigan with the intention of completing a full classical course of study. But frequent interruptions from ill health finally caused him to relinquish this course at the end of the sophomore year, and to turn his attention to ordinary business pursuits. In a few months, however, he returned to the office of his father and commenced a dilligent study of medicine in the spring of 1867. In the autumn of the same year he matriculated in the Chicago Medical College, and faithfully pursued the systematic graded course of that institution until near the close of his second college year, when, on account of some threatening pulmonary symptoms, he went to Europe, where he remained sixteen months. While absent he continued to devote a part of his time to the study of medicine, a part to the acquisition of a more practical knowledge of the German and French languages, and the rest to general science and observation. On his return home he immediately resumed his regular course of study in the Chicago Medical College and Mercy Hospital, and graduated at the end of his third college year, in March, 1871; receiving the prize for the best thesis presented to the faculty by the class of that year. Immediately after graduation he again visited Europe, accompanied by his younger brother, and spent a few months in the hospitals of Vienna, giving more special attention to the diagnosis and treatment of diseases of the air passages. Returning home the latter part of July of the same year, he entered at once upon the general practice of his profession in this city. Invigorated by his two trips to Europe, he was enabled to pursue the practice of his profession, uninterruptedly and with a gratifying degree of success, until June 3, 1880, when he was suddenly arrested by the sickness that ended in his death at the date already given. During this brief professional career of only nine years, he had not only laid the foundation for a lucrative practice and attached to himself the fullest confidence and affection of a wide circle of friends, but he had also equally won the respect and esteem of the members of his own profession. As proof of the latter, and also as an indication of his activity in the promotion of both the social and scientific interests of the profession it may not be inappropriate to mention that he was an active member of the American Medical Association; the Illinois State Medical Society; the Alumni Association of the Chicago Medical College; the Chicago Medical Society; the American Laryngological Association, and the Association of American Medical Editors, to the transactions of all of which he contributed papers of more or less value, and in the three last of which he held official positions. He was also an active member of the medical staffs of the Mercy Hospital and of the South Side Free Dispensary. Soon after entering the profession he became associated with his father as editor of the CHICAGO MEDICAL EXAMINER, which position he continued to hold until that periodical was transferred to the Chicago Medical Press Association, and united with the CHICAGO MEDICAL JOURNAL. Yet in the midst of all his professional engagements he found time to cultivate the broader domain of general science, and early became an active member of the Chicago Academy of Sciences, of which he was Librarian at the time of his death. If we turn for a moment from his professional career to his private and domestic character, we shall find in it nothing but comfort and consolation to the sorrowing ones he has left behind him. Naturally timid, sensitive and affectionate, he early imbibed a profound respect for the precepts and doctrines of the Christian religion, and after arriving at mature years, became an acceptable member of the Methodist Episcopal Church; and though some portions of his last illness were accompanied by the most intense bodily suffering, yet no word of murmuring against Providence escaped his lips. For three weeks before his death he became fully conscious of the probable fatal result and expressed his readiness to go, with the utmost confidence in his future welfare. On the 13th of April, 1875, he was united in marriage to Miss Anna Smith Marcy, the accomplished daughter of Prof. Oliver Marcy, of Evanston, the acting president of the Northwestern University, and entered at once upon the enjoyment of that domestic happiness which can be found only in a home of virtue, intelligence and affection. In due time two children, a son and daughter, were added to the family circle, and, with the mother, are now left to mourn their great loss. The chief cause of death, as shown by a post mortem examination, was an acute pyæmic or suppurative inflammation of the left kidney, accompanied by so persistent a reflex irritation of the stomach, that during the last three weeks of life, his sole dependence for support was on nutritive enemas. The deceased was an industrious student; a careful and accurate investigator; a ready writer; with a rare mechanical genius and fine appreciation of art. The latter qualities were displayed in the devising and modifying of instruments for his own use in the favorite department of his profession, and in the modelling of a very accurate life size bust of his father.

---

TO REMOVE PLASTER OF PARIS FROM THE HANDS.—Dr. Wilcox, surgeon, U. S. A., says that a little bicarbonate of soda or potassa, added to the water in which the hands are washed, after applying plaster of Paris bandages, immediately removes the unpleasant feeling left by the plaster.—*Toledo Med. and Surg. Journal.*

